# Exploring the influence of the home literacy environment on early literacy and vocabulary skills in Korean–English bilingual children

**DOI:** 10.3389/fpsyg.2024.1336292

**Published:** 2024-03-08

**Authors:** Wi-Jiwoon Kim, Dongsun Yim

**Affiliations:** Department of Communication Disorders, Ewha Womans University, Seoul, Republic of Korea

**Keywords:** heritage language, bilingualism, home literacy environment, early literacy, vocabulary

## Abstract

Studies have emphasized the significance of maintaining a heritage language for various reasons such as the establishment of linguistic and cultural identity, as well as socio-emotional development. Despite the crucial role that literacy development in a heritage language plays in language preservation, there is a scant research that explores the impact of home literacy environment and literacy development in children with a heritage language. This study aimed to examine the home literacy environment and literacy-related skills in 4-to 5-year-old Korean–English bilingual children living in an English-speaking country, Australia, whose heritage language is Korean, and to investigate the relationships among the home literacy environment factors and the child-internal literacy-related skills. The study employed parental questionnaires and video analyses of parent–child shared book reading sessions to assess the Korean and English home literacy environment. Children’s early literacy skills in Korean and English, along with their Korean, English, and conceptual vocabulary skills, were measured as literacy-related skills. The findings indicated that parents utilized an indirect approach for Korean literacy practices, in contrast to a more direct and explicit method for English literacy practices. However, active and direct literacy practices were found to be essential for Korean early literacy development, while indirect methods are sufficient for English early literacy skills. Moreover, the availability of abundant Korean literacy resources at home had a positive impact on the development of Korean and English, as well as conceptual vocabulary skills. In conclusion, this study underscores the importance of providing a robust literacy environment in a heritage language in bilingual families to promote language proficiency in both the heritage language and the dominant social language, while also supporting the development of conceptual language skills.

## 1 Introduction

Preserving a heritage language, particularly when it is regarded as a minority language with lower social status compared to the dominant language in a society, presents a formidable challenge. A study conducted by Baratz-Snowden et al. (1988) examined the self-assessed language proficiency of children from immigrant families in the U.S. The results revealed that children whose parents had immigrated from Asian countries had significantly higher proficiency in English, the dominant social language, than in their heritage language. Even when immigrant families continue using their heritage language within their homes, the pervasive societal prioritization of the dominant language imposes substantial obstacles to children’s heritage-language fluency. Consequently, children within these families often encounter communication difficulties with family members, such as their grandparents (Cho and Krashen, 1998).

Nonetheless, individuals should preserve their heritage language to facilitate effective communication with their family members, thereby validating and strengthening their cultural and linguistic identity, which ultimately affects their socio-emotional development (Cummins, 1981). Previous research has revealed that within multicultural families in Korea, which consist of Korean fathers and immigrant mothers, adolescent children who used their mothers’ native language at home demonstrated significantly higher levels of self-esteem, ego resilience, referring to an individual’s ability to adapt to adverse situations without experiencing psychological breakdown, bicultural and academic adaptation, and more positive relationships with their teachers than their counterparts who exclusively communicated in Korean at home (Song and Yim, 2020). These internal socio-emotional factors might affect children’s adaptation to school. Among children in immigrant families in the U.S., those who only spoke English experienced significantly higher dropout rates in schools than their peers who maintained their heritage language (Feliciano, 2001).

Immigrant parents also demonstrate a clear understanding of the importance of preserving their heritage language. Korean immigrant parents in the U.S. recognize the significance of their children maintaining their heritage language, Korean. They are well aware of its practical career-related, cognitive, and emotional advantages, along with its role in establishing cultural identity and preserving cultural heritage. Moreover, they are willing to engage in bilingual programs to facilitate their children’s use of the Korean language (Shin and Krashen, 1998). In Canada, Korean immigrant parents similarly emphasize the importance of maintaining the heritage language, expressing high expectations regarding their children’s proficiency in Korean. However, they prioritize the development of comprehension and speaking skills over reading and writing skills (Park and Sarkar, 2007).

While the emphasis on oral language skills often overshadows literacy development in a heritage language, successful literacy development remains a vital component in preserving the language for individuals. Literacy, encompassing reading and writing proficiencies, plays a pivotal role in intellectual and emotional growth, shaping positive personalities, and broadening individual’s perspectives through vicarious experiences with text (Palani, 2012). Consequently, among children in immigrant families, literacy education in a heritage language holds significant value in cultivating a deeper understanding of and positive attitude toward their language and culture. Previous research has shown that children with strong literacy skills in their heritage language are more likely to maintain their proficiency in spoken language, and literacy education contributes to the preservation and growth of the heritage language (Chevalier, 2004; Lee, 2012; Leonard et al., 2020). Exploring the factors that influence literacy skills in a heritage language and their relationship with child-external factors is critical to enhancing those skills among children in immigrant families.

### 1.1 Literacy development

Literacy skills primarily develop through explicit instruction in schools. However, implicit learning, such as statistical learning which involves the ability to detect and internalize statistical patterns and regularities in the environment, also plays a fundamental role in the development of literacy. In the context of reading skills, Apfelbaum et al. (2013) discovered that children could extract information regarding grapheme-phoneme correspondence regularities through repeated exposure over several days without explicit teaching in a school setting. Moreover, numerous studies have established a connection between developmental dyslexia and implicit learning mechanisms, indicating that children’s reading skills are influenced by environmental exposure and internal factors (Vicari et al., 2003, 2005; Gabay et al., 2015). Researchers have suggested that writing skills, especially general spelling patterns, are also influenced by implicit learning (Pollo et al., 2009; Treiman et al., 2018; Zhang and Treiman, 2021). Treiman and Kessler (2006) demonstrated that children can discern orthographic patterns from their surrounding environment without explicit instruction by observing children’s spelling patterns with pseudowords, where, for instance, children were more inclined to write pseudowords using letter combination <ea> when /ɛ/ is followed by the coda /d/.

The development of literacy is influenced not only by environmental factors that provide exposure related to literacy but also by various essential skills in children. The direct and indirect effects model of reading (DIER; Kim, 2017, 2020a,b) effectively explains how the skills involved in reading comprehension hierarchically interact with each other. This model includes proximal skills that are directly associated with reading comprehension and distal skills that provide indirect support to proximal skills, such as domain-general executive functions, thereby impacting reading comprehension. Proximal skills include word reading, which relies on early literacy skills, and listening comprehension, which is based on foundational language skills like vocabulary.

Early literacy skills encompass several key components, including phonological awareness, letter name knowledge, and orthographic knowledge. These components have been consistently identified as critical predictors of children’s subsequent literacy development, applicable in both Korean and English contexts (Kim, 2011; Kim et al., 2013). Phonological awareness, which refers to the capacity to recognize, discriminate, and manipulate the sounds within a language (Anthony and Francis, 2005), lays the foundation for reading processes such as decoding, blending, and, ultimately, word reading. It also stands out as the most robust predictor of development in reading, and this pattern has been observed universally across diverse alphabetic languages (Melby-Lervåg et al., 2012). Letter name knowledge serves as a significant predictor of literacy development, indicating the commencement of phonological processing of print and facilitating the acquisition of letter-sound relationships and the development of phonemic sensitivity skills (Wagner et al., 1997; Noel Foulin, 2005). Orthographic knowledge pertains to the stored information in one’s memory that facilitates precise writing in the orthography of a specific language (Apel, 2011). The acquisition of orthographic knowledge makes a distinctive contribution to children’s reading and writing skills by enabling them to quickly and accurately recognize words, thus enhancing text comprehension (Ehri, 1992; Conrad et al., 2013; Querido et al., 2021).

Both word reading and listening comprehension are developed within the child’s surrounding environment (Kim, 2023). Therefore, for a better understanding of and support for children’s literacy development, it is crucial to comprehend the interplay among children’s internal factors, encompassing early literacy and general language competencies, and external factors.

### 1.2 Home literacy environment

Children are exposed to a multitude of literacy experiences in their daily lives through various forms of print, such as signs, billboards, labels, books, and more. The home literacy environment (HLE) is especially renowned for its critical influence on children’s literacy development. The HLE is a multifactorial construct encompassing a wide range of experiences related to reading and writing. These include interactions between adults and children during reading and writing activities, a child’s independent exploration of written materials, and a child’s emulation of literate behaviors exhibited by adults (Teale and Sulzby, 1986). Furthermore, considering the impact of media consumption, such as television viewing or gaming, on children’s language and literacy development in current society, taking into account both media exposure and print exposure is essential (Uchikoshi, 2005; Dixon, 2011). Previous studies have shown a direct relationship between the extent of parent–child engagement in literacy and language activities at home and children’s literacy and language skills, as well as the positive impact of interventions designed to enhance the quality of the HLE on children’s linguistic competencies, including vocabulary skills (Payne et al., 1994; Griffin and Morrison, 1997; Niklas and Schneider, 2015; Napoli and Purpura, 2018).

Sénéchal and LeFevre (2002) introduced a model that classifies the HLE based on whether the learner’s at-home literacy practice is formal or informal. Formal literacy practice (FL) centers on the form of written language and the letter-sound relationship, involving activities such as reading alphabet books to the learner or directly teaching them letter sounds. In contrast, informal literacy practice (IL) encompasses activities where the primary emphasis is on the message conveyed by the printed text, rather than solely on the physical characteristics or sound of the text, which include a range of activities related to exposure to print and media. It has been suggested that FL and IL may distinctly influence children’s literacy development. FL is particularly crucial for written language skills, fostering early literacy skills and initial efforts in reading and writing, whereas IL, such as exposure to print and media and storybook reading, is more associated with overall oral language proficiency (Anderson, 1995; Sénéchal et al., 1998; Sénéchal and LeFevre, 2002).

Literacy exposure often begins during the early stages of a child’s development with the exploration of books. The availability of abundant literacy resources, such as the number of books in households, continues to significantly influence children’s reading fluency and early literacy skills even after considering parental and children’s intelligence factors (Raz and Bryant, 1990; van Bergen et al., 2017). Moreover, frequent interactions involving print exposure between parents and children are related to early literacy and vocabulary development (Payne et al., 1994; Sénéchal et al., 1998; Richman and Colombo, 2007; Kim et al., 2022). Sénéchal et al. (2008) examined the relationship between the frequency of shared book reading and early literacy and language skills in four-year-old children. The findings indicated that frequent shared reading predicts children’s expressive vocabulary and morphological knowledge, even when accounting for children’s nonverbal intelligence and parental factors such as socioeconomic status and reading proficiency.

The impact of media exposure on children’s language and literacy development remains controversial. Previous research has indicated that the effects of media exposure may vary based on the age of the children and their environmental background. For instance, children who are approximately 5 years old, those from lower socioeconomic backgrounds, or those with limited language skills may experience some benefits from television viewing in terms of language and literacy development, including improvements in vocabulary, reading achievement, and academic performance (Searls et al., 1985; Rice et al., 1990; Comstock and Paik, 1991; Wright et al., 2001). However, for children younger than 3 years old, exposure to media may be disadvantageous (Taylor et al., 2018).

Parent–child shared book reading is critical to literacy development because it enables simultaneous engagement in FL and IL, making it one of the most effective methods for facilitating children’s language and literacy development. While reading books with their children, parents often participate in various formal and informal literacy activities, including reading the text aloud to help children learn to read certain words and discussing the content of the book, respectively. Furthermore, book reading has gained significant attention since it provides scaffolding effects, as suggested by Vygotsky (1978), on children’s speech production. It also offers parents a rich source of vocabulary that they can employ when communicating with their children (Hoff-Ginsberg, 1991; Hoff, 2010). In the context of book reading, supportive actions through various reading strategies are taken by adults to help children understand a text and expand their knowledge related to language and literacy, building upon their existing skills.

In addition to frequency, the quality of interaction during shared book reading also plays a crucial role in children’s language development. Kim et al. (2022) investigated the connection between the home literacy environment, specifically shared book reading, and the language skills of children aged four to six. They discovered that frequent and repetitive book reading was positively associated with children’s expressive vocabulary. Furthermore, the interactive book reading style employed by parents, which encourages active participation from children during shared book reading through activities like asking open-ended questions, was found to be related to children’s receptive vocabulary. Studies about parental book reading interventions demonstrate the impact of book reading interactions on children’s language and literacy skills. In a control study by Whitehurst et al. (1988), the group that received a book reading intervention focused on specific reading strategies, such as using “what” questions to encourage discussion, expanding or correcting the child’s speech, and adapting to the child’s developmental level and interests showed significant improvements in expressive vocabulary compared to the control group that did not receive the intervention. A 12-week intervention conducted by Newman (1996) that aimed at enhancing interactions during shared book reading resulted in improvements in children’s receptive vocabulary skills and early literacy skills, such as print concepts, irrespective of their parents’ literacy proficiency.

In summary, when examining the HLE and its impact on children’s language and literacy development, FL and IL must both be considered as their influences on various domains of children’s language and literacy skills may differ. FL is widely recognized for its role in early literacy development, while IL, which includes frequent exposure to print and media, can affect language and literacy skills, albeit with some debate surrounding the effects of media exposure. Parent–child shared book reading is of particular significance in this context since it provides an opportunity to engage in both FL and IL. Moreover, the quality of these interactions during shared book reading is closely linked to the development of expressive and receptive vocabulary skills, as well as early literacy skills.

### 1.3 Cross-linguistic effects in bilingual home literacy environments

Individuals with a heritage language experience bilingualism since they are required to employ at least two languages in their daily communication, where they use their heritage language with family members and a social language at school, work, or within their local community, regardless of their fluency in either language (Grosjean, 2008; Kohnert, 2013).

Bilingual individuals may experience different aspects of bilingualism depending on the difference in social power that each language holds: additive and subtractive bilingualism (Lambert, 1981). Additive bilingualism happens when one’s home language shares comparable social status with the dominant social language. For example, Korean-English bilingual children living in Korea acquire their second language, English, while maintaining their first language, Korean, since English is well-regarded in society. On the other hand, subtractive bilingualism often affects ethnolinguistic minority communities, such as Korean families living in the U.S., whose heritage language (Korean) has weaker power than the social language (English). In such cases, there are fewer opportunities to communicate in their first language within the larger community, leading to their first language not being reinforced throughout the lifespan of learning the second language. Thus, it is necessary to consider the power relationship between two languages when discussing language development in bilingual children.

In the context of bilingualism, language transfer occurs when elements from one language affect the usage of another language in bilingual individuals. This phenomenon has been documented across various linguistic domains, including phonology, semantics, pragmatics, and others (Kasper, 1992; Durgunoğlu et al., 1993; Atwill et al., 2007; Rasier and Hiligsmann, 2007).

Bilingual children experience distinct HLEs compared to their monolingual peers in terms of the quantity and quality of language input in each language. Additionally, research suggests that bilingual individuals may differ from monolinguals in how they utilize available resources to enhance their reading abilities (Peets et al., 2019). Therefore, when examining the HLE within bilingual populations, the dynamic relationships between the HLE and linguistic skills must be investigated for both languages, as these factors are intricately intertwined and influence each other. Despite the unique features of the HLE and language development in bilingual children, studies on the relationship between these aspects are scarce. Moreover, only a limited number of studies have taken both languages into account when examining this relationship.

Ryan (2021) investigated the relationships between print and media exposure (PME) and vocabulary development in English and French among English–French bilingual children in the early elementary school years in the U.S. The findings revealed that English PME did not significantly impact their English vocabulary, but French PME positively influenced their French baseline vocabulary. The language used at home played an essential role in French vocabulary development but not English vocabulary development. This implies that engaging in home literacy activities in French is crucial to support the growth of their French vocabulary in an English-speaking country. Although both languages were considered, this study had limitations in that the HLE was assessed solely based on PME. Farver et al. (2013) extensively explored the relationship between the HLE and language skills in both languages, encompassing a wide range of factors, including parents’ literacy habits, home literacy resources, parental literacy practices assessed through questionnaires and interviews, and an evaluation of children’s cognitive abilities, oral language skills, phonological awareness, and print knowledge. Spanish–English bilingual children aged 41 to 60 months and their parents living in the U.S. participated. Significant positive correlations were found among early literacy and language skills, with the exception of Spanish expressive language skills in relation to English oral language and phonological awareness. Language-specific and cross-linguistic relationships among the HLE factors were observed, but Spanish and English HLE factors tended to exhibit negative correlations. A Spanish HLE was negatively correlated with children’s English oral language and phonological awareness, while parental factors within a Spanish HLE positively influenced children’s Spanish oral language skills and print knowledge. These findings may not signify a detrimental effect of HLE on heritage language skills. Instead, they might be influenced by a subtractive bilingual environment and parental attitudes or priorities regarding literacy education. Previous research has shown that engaging in heritage-language literacy activities at home does not negatively affect bilingual children’s second language development (Dekeyser and Stevens, 2019; Sun et al., 2023).

In the context of book reading within bilingual families, parents may utilize similar reading strategies as monolingual parents (Boyce et al., 2004). However, they often exhibit distinctive characteristics in their reading approaches, such as code-switching, which involves switching to another language during story discussions or translating words to introduce new vocabulary (Gonzalez-Barrero et al., 2021; Yang et al., 2021). Recent research has highlighted the positive impact of frequent shared book reading in bilingual children’s heritage language on their receptive vocabulary in that language during the preschool years (Sun et al., 2023). Therefore, it is also crucial to consider how parents employ each language when reading books in different languages within bilingual families, emphasizing the importance of examining the HLEs in both languages.

### 1.4 Present study

This study aimed to comprehensively examine the relationships between the HLE in each language and literacy-related skills in each language among Korean–English bilingual children who primarily use Korean as a heritage language and English as a dominant social language. This study employed a multifaceted approach to assess the HLE. Questionnaires were administered to measure quantitative and qualitative aspects of PME, FL, and IL that occur in both Korean and English. Furthermore, the study analyzed actual parent–child shared book reading sessions with both Korean and English books. Additionally, the foundational literacy skills in both languages, which are critical predictors of future reading and writing abilities such as phonological awareness, letter name knowledge, and orthographic knowledge, alongside vocabulary skills in Korean, English, and conceptual domains, were examined.

## 2 Methods

### 2.1 Participants

A total of 36 typically developing Korean–English bilingual children aged 4–5 years (mean age 59.1 months, SD = 6.69), along with their parents, residing in an anglophone country, Australia, participated in this study. The selection of the 4-to 5-year-old age range was based on previous research indicating the development of fundamental literacy skills in children of this age prior to formal education, which typically commences in the first grade (Kim, 2010). The primary caregivers of the children spoke Korean as their native language, and all participants primarily used Korean at home while employing English for everyday communication. All children achieved scores above the 10th percentile on both the Korean Receptive & Expressive Vocabulary Test (REVT; Kim et al., 2009) and English Expressive One-Word Picture Vocabulary Test-4 (EOWPVT-4; Martin and Brownell, 2010) and Peabody Picture Vocabulary Test-4 (PPVT-4; Dunn and Dunn, 2007) when conceptual scoring was implemented. All children scored above 85 in standard scores of nonverbal intelligence, assessed using the Korean Kaufman Brief Intelligence Test-2 (Moon, 2020), and no physical, sensory, or neurological difficulties were reported by parents.

All child–parent dyads read a Korean book and an English book, each with different content. Four book reading sets were created based on the language of the book (Korean or English) and the content of the book (book A or book B): set 1 (Korean book A—English book B), set 2 (English book B—Korean book A), set 3 (Korean book B—English book A) and set 4 (English book A—Korean book B). Each child–parent dyad was randomly assigned to one of these sets to minimize the effect of book familiarity and counteract potential order effects. Each set had 9 dyads, and one-way analysis of variance (ANOVA) tests revealed no significant differences in age, vocabulary, or nonverbal intelligence among these sets [age: *F*(3, 32) = 0.98, *p* = 0.461; REVT (expressive): *F*(3, 32) = 0.59, *p* = 0.626; REVT (receptive): *F*(3, 32) = 0.42, *p* = 0.742; EOWPVT-4: *F*(3, 32) = 0.88, *p* = 0.464; PPVT-4: *F*(3, 32) = 0.71, *p* = 0.554; KBIT-2: *F*(3, 32) = 0.18, *p* = 0.912]. The characteristics of children in this study are presented in Table 1.

**Table 1:** Children’s characteristics.

*N*	Age (month)	REVT (expressive) (raw score)	REVT (receptive) (raw score)	EOWPVT-4 (raw score)	PPVT-4 (raw score)	KBIT-2 (standardized score)
36 (*F* = 20, *M* = 16)	59.1 (6.69)	58.72 (9.43)	70.92 (12.24)	65.83 (9.98)	90.72 (17.49)	113.89 (17.70)

Values are presented as mean (SD); All vocabulary test scores reported are outcomes from conceptual scoring. REVT, Receptive & Expressive Vocabulary Text (Kim et al., 2009); EOWPVT-4, Expressive One-Word Picture Vocabulary Test-4 (Martin and Brownell, 2010); PPVT-4, Peabody Picture Vocabulary Test-4 (Dunn and Dunn, 2007); KBIT-2, Korean Kaufman Brief Intelligence Test-2 (Moon, 2020).

### 2.2 Measures

#### 2.2.1 Parental questionnaires

Parents completed 6-page paper questionnaires that covered the number of Korean and English books they possessed in their homes, the frequency of print and media exposure (PME) in each language for their children at home, and the extent of formal literacy practice (FL) and informal literacy practice (IL) they engaged in with their children at home in each language. The questionnaires were provided in Korean, taking into account the parents’ native language.

PME was assessed according to the PME questionnaire developed by Ryan (2021). Parents were instructed to evaluate their child’s exposure to print and media materials at home, outside of school, in both Korean and English. The questionnaire included activities such as reading, watching television or movies, playing games, and listening to songs.

Regarding FL, the questionnaire created by Skwarchuk et al. (2014) was utilized. Parents were asked about how often they engage in FL with their children at home in Korean and English. The questionnaire broadly covered identifying, reading, or teaching letters, words, and sound-letter relationships. One item in the questionnaire, “We make up rhymes in songs,” was adapted for the Korean language to “We play with the sounds of the letter in songs.” Since in Korean, the body-coda structure is more salient than the onset-rhyme structure (Cho and McBride-Chang, 2005), it was not applicable to Korean.

For IL, the adapted version of the home literacy environment questionnaire (Kim et al., 2022) was employed. Generally, IL encompasses a variety of activities related to exposure to print and media. However, in this study, informal literacy practices were limited to those occurring during parent–child book reading activities, distinguishing them from the abundance of literacy resources and the frequency of print and media exposure. Parents were requested to indicate the frequency with which they participated in informal literacy practices with their children while reading Korean and English books. The informal literacy practices encompassed in the questionnaire were talking about the content of the book the child shows interest in, asking questions about the book to ensure their understanding or make them guess what would happen next, connecting the content of the book to their daily lives, and so on.

All questions, except the question about the number of books in the household, were rated on a 5-point Likert scale: 1 = “never,” 2 = “rarely (i.e., every once in a while, but not every week),” 3 = “sometimes (i.e., once or twice a week),” 4 = “often (i.e., more than twice a week, but not every day),” 5 = “always (every day).”

#### 2.2.2 Parent–child shared book reading

The primary caregivers participated in the shared book reading sessions with their children, involving one Korean and one English book. In instances where both parents were considered primary caregivers, those who mainly read to their children were encouraged to participate in the session. Parents were required to read the books with their children as usual. Each book reading session was videotaped for 10 min, a duration determined based on the time it took participants to complete each book in a preliminary study. If a book reading session, including post-reading interactions, concluded in less than 10 min, the entire session was considered for analysis.

Two Korean and two English books, all age-appropriate for the participants, were selected for the parent–child shared book reading sessions. The English book reading session featured “If I Built a House” by Chirs Van Dusen (2012) (book A) and “If I Built a Car” by Chris Van Dusen (2005) (book B). For the Korean book reading session, the Korean-translated versions of each book, translated by Sarang Yu, were used. While books A and B had different main topics, they shared a similar structure and flow. Korean books A and B contained 693 and 830 words, respectively, averaging 761.5 words. English books A and B contained 659 and 762 words, respectively, averaging 710.5 words.

##### 2.2.2.1 Text-read ratio and Korean interactive utterance ratio

The total number of words spoken by parents was counted, and the text-read ratio (Tr) and Korean interactive utterance ratio (Kr) were calculated. The Tr was determined by dividing the total number of words in the text read by the parent by the total number of words in the book’s text and then multiplying by 100. The Kr was considered for analysis since the parents in this study were Korean–English bilinguals, although their native language was Korean. The ratio allowed for an examination of how the amount of language usage in different languages was influenced by the book stimuli and its impact on children’s literacy-related skills. The ratio was calculated by dividing the total number of Korean words spoken by the parent during interactive conversation while reading the book by the total number of words in all interactive utterances by parents and then multiplying by 100. Words were counted instead of morphemes due to differences in linguistic properties between Korean (an agglutinative language) and English (an analytic language with inflectional aspects). Comparing Korean and English utterances based on morpheme counts would introduce bias against English, as English typically contains fewer morphemes per word compared to Korean. The employment of word counts is more suitable for the comparison between the two languages on a semantic level.

##### 2.2.2.2 Parental book reading strategies: formal and informal literacy practices

Parental book reading strategies, encompassing both formal and informal literacy practices, were analyzed during the parent–child shared book reading sessions. Interactive utterances were assessed using frameworks developed by DeTemple (1994) and Haden et al. (1996). They were categorized to distinguish between formal and informal literacy practices based on their interactive characteristics. Utterances categorized as reading strategies included interactive statements and questions, provided that they were intended to elicit responses aligned with the goals of the parental book reading strategies. When multiple successive utterances shared the sample topic and strategy, they were counted as a single instance. Excluded from the analysis were utterances such as statements or questions unrelated to the book’s content, simple repetitions of their children’s utterances, basic responses to children’s questions (e.g., “Yes, it is”), simple exclamations (e.g., “Wow, it’s wicked!”), statements of questions capturing children’s attention (e.g., “Look at this,” “What is it?”), and questions checking children’s comprehension (e.g., “Did you get it?”). Formal and informal literacy practices were coded according to the criteria outlined in Table 2. Formal literacy practices had three strategies: letter/word related reference, letter-sound relationship, and definition of the word; informal literacy practices encompassed six strategies: simple description, elaborate description, links to the world, prediction inferences, text recall/recitation, and book concepts. After coding the utterances, the total number of strategies was computed.

**Table 2:** Formal and informal literacy practices in book reading strategies.

Literacy practice type	Strategy	Definition	Example
Formal literacy practice	Letter/word-related reference	Focusing the child’s attention on certain words or letters in the text, occasionally accompanied by verbalization of support with the child’s reading	Pointing at a word and enunciating it
	Letter-sound relationship	Guiding the child on the correspondence between letters and sounds	Rhyming words, teaching the sounds associated with specific letters
	Definition of the word	Providing the meanings of words in the book	“[while gesturing] Dome is like this round structure of the building,” Translating difficult English words into familiar Korean equivalents, or vice versa.
Informal literacy practice	Simple description	Providing basic explanations of physical traits of people, objects, actions, locations, and comparable elements encountered in the book	“This car looks so round,” “What is this?”
	Elaborate description	Detailing the events described in the book, providing a comprehensive explanation of the book’s content, rephrasing complex expressions without aiming to teach word definitions, and exploring elements that are hinted at in the illustrations but not explicitly mentioned in the text	“It’s in Jack’s imagination,” “So this car does not make any sound unlike other cars,” “I think here’s an elevator.”
	Links to the world	Creating links between the storyline and real-world events or personal experiences	“This looks just like the car wash we visited last month.” “What house would you build if you could build a house?”
	Prediction inferences	Anticipating future developments in the story and conjecturing about characters’ motives, inner thoughts, or cause-and-effect relationships	“What will come next?” “These people must’ve been so shocked because they’d never seen something like this before!”
	Text recall/recitation	Reciting parts of the book’s text from memory without re-reading the exact text and translating the text from memory without the intention of teaching word meanings	
	Book concepts	Discussing the book’s attributes, including the title, author, illustrator; page-turning; or the act of reading itself	“Turn to the next page,” “This page was written to say thank you to the author’s parents.”

##### 2.2.2.3 Parental sensitivity

Parental sensitivity (PS) was assessed using the MULTI-PASS (Marfo, 1992) video coding scheme, which is designed to analyze parent–child interactions, to gauge the social–emotional aspect of interactions during the parent–child shared book reading sessions. This coding scheme evaluates six dimensions of parental behavior: warmth, sensitivity, responsiveness, encouragement of initiative, stimulation value, and elaborateness (see Table 3). Each of these dimensions was rated on a 5-point Likert scale, with 1 indicating the lowest level of the coded behavior and 5 the highest. Two coders participated in the rating process, and the average scores of each behavior between the coders were calculated. PS was determined by computing the average scores across the six behaviors.

**Table 3:** Parental interactive behaviors evaluating parental sensitivity.

Interactive behavior	Definition
Warmth	The parent’s display of positive emotions towards the child, which may include affectionate verbal expressions and physical gestures that convey fondness
Sensitivity	The parent’s ability to accurately interpret and effectively respond to both verbal and nonverbal cues exhibited by their child, as well as their awareness of the child’s developmental capabilities
Responsiveness	The parent’s reaction to the child’s interests and observable behaviors in a timely, consistent, and appropriate manner
Encouragement of initiative	The parent’s interaction style that acknowledges the child’s need for independence and self-direction, which includes promoting decision-making and encouraging exploration during book reading
Stimulation value	The parent’s capacity to offer cognitive or linguistic stimulation to the child, actively seeking opportunities to improve the child’s cognitive or linguistic skills
Elaborateness	The parent’s actions of elaborating on or expanding upon the verbal and nonverbal cues displayed by the child during their interaction

#### 2.2.3 Children’s early literacy and vocabulary skills

Children’s early literacy skills encompassed phonological awareness, which included syllable awareness (SA) and phoneme awareness (PA), letter name knowledge (LN), and orthographic knowledge (OK). Each of these skills was assessed in both Korean and English.

This study employed a modified version of the Korean phonological awareness tasks, adapted from Kim and Pae (2007) and Jung et al. (2015). For the English phonological awareness tasks, a similar format to the Korean phonological awareness tasks was created for this study. Prior to the tasks, two practice items in each test section were provided to ensure the children’s understanding of the task. The syllable awareness tasks consisted of ten items in syllable counting (e.g., How many times can you clap for the word “computer?”), ten items in syllable blending (e.g., What word can you get from these sounds? “ro,” “bot”), five items each in first syllable and last syllable deletion (e.g., What is “person” without “per”? What is “basket” without “ket”?), and five items each in first syllable and last syllable discrimination (e.g., Among these words, which one starts with a different sound? “tickle,” “ticket,” “lonely.” Among these words, which one ends with a different sound? “raccoon,” “bedroom,” “cocoon”). The phoneme awareness tasks encompassed five items each in onset-body blending and body-coda blending (e.g., What word can you get from the sounds /b/ and /ig/? What word can you get from the sounds/pi/and/ck/?), five items each in initial and final phoneme deletion (e.g., What is “bed” without /b/? What is “goat” without/t/?), and five items each in onset and coda awareness in Korean, and onset and rime awareness in English (Among these words, which one starts with the different sound? “big,” “bat,” “down.” Among these words, which one ends with a different sound (Korean coda awareness)? “Bob (밥), Jib (집), Kong (콩).” Among these words, which one ends with a different sound (English rime awareness)? “house,” “mouse,” “cat”). In the Korean and English letter name knowledge tasks, children were instructed to verbally state the name of each letter on the screen. A total of 40 Korean letters, including 19 consonants and 21 vowels, were presented individually in a random order. In the English letter name knowledge task, a total of 26 English letters were randomly presented on the screen one by one, with both uppercase and lowercase letters displayed simultaneously.

In this study, the orthographic choice task by Wang et al. (2006) was adapted for the Korean orthographic knowledge test, and the word-likeness tasks from Conrad et al. (2013) were used for the English orthographic knowledge test. The Korean orthographic task was developed based on six constraints specified by Kim (2011). Children were presented with one legal letter that is the real letter and one illegal letter that does not follow one of these constraints, displayed on the same screen, and then asked to identify the real letter. The test consisted of a total of 26 items, including two practice items and four items targeting each of the six constraints. The six constraints targeted in the test are as follows: (1) vowel placement constraint, dictating that a horizontal vowel should be positioned below the onset consonant and a vertical vowel should be placed to the right side of the onset consonant or a combination of an onset consonant and a horizontal vowel (e.g., 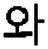 is legal, but 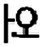 is illegal); (2) complex vowel legality concerning the permissibility of combining certain vowels with other specific vowels (e.g., 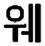 is legal, but 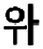 is illegal); (3) onset consonant combination constraint, which prohibits the placement of certain consonant combinations in the onset position (e.g., 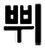 is legal, but 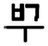 is illegal); (4) coda consonant combination legality, pertaining to the allowance of specific consonant combinations in the coda position (e.g., 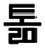 is legal, but 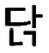 is illegal); (5) legal double consonants in coda, specifying that only certain double consonants are allowed in the coda position (e.g., 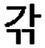 is legal, but 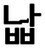
is illegal); and (6) mandatory onset letter requirement, indicating that every syllable must contain a letter in the onset position (e.g., 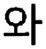 is legal, but 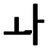 is illegal).

For the English orthographic knowledge test, a pair of 4-letter homophonic pseudowords was presented on the screen, and the examiner pronounced the words. Children were asked to point to the word that seemed more like a real word in English. The homophones were created (1) by having letters at the end sharing the same sound (e.g., “lunk/lunc”), (2) using commonly used long vowels alongside non-common long vowels or combinations of two non-common vowels (e.g., “nide /nyde,” “dake/daik”), and (3) using commonly used vowel digraphs alongside non-common vowel digraphs or less common long vowels in the context (e.g., “moin/moyn,” “poaf/pofe”). A total of 26 items were provided, including two practice items and eight items in each type.

For the vocabulary skill assessments, the REVT (Kim et al., 2009) was administered to evaluate expressive and receptive Korean vocabulary in children, and the EOWPVT-4 (Martin and Brownell, 2010) and PPVT-4 (Dunn and Dunn, 2007) were utilized to assess their expressive and receptive English vocabulary skills, respectively. When applying conceptual scoring, the translated versions of the REVT (receptive) and PPVT-4 provided by Yim et al. (2022) were utilized. The translation of the REVT (expressive) and EOWPVT-4 was carried out by two graduate students majoring in Communication Disorders. Each translated version underwent a thorough review process involving two native English speakers with a minimum of 5 years of work experience in daycare centers in Canada or Australia, as well as two native Korean speakers holding a Korean Speech-Language Pathologist License. Following this review process, the final transcripts were validated by a bilingual speaker proficient in both Korean and English, holding a Speech-Pathology Australia practicing certificate. Conceptual expressive and receptive vocabulary skills were evaluated using the Korean and English versions of vocabulary tests for language screening. However, for the analysis, results of conceptual vocabulary assessed with the EOWPVT-4 and PPVT-4 were considered since they were deemed to be a more culturally relevant evaluation of their vocabulary skills, taking into account that the children reside in Australia. The REVT was originally designed to assess Korean-speaking children in Korea, which includes cultural aspects specific to Korea.

In the expressive part of the REVT, a single picture was presented, and the children were asked to say the corresponding word in Korean. Once the ceiling level was reached, the examiner reintroduced pictures of items that had been answered incorrectly and asked the children to provide the English word to evaluate their conceptual expressive vocabulary. Similarly, in the EOWPVT-4, the children were initially instructed to express the words in English; if their responses were incorrect, they were prompted to provide the Korean equivalents. In the receptive part of the REVT, the examiner displayed four pictures and instructed the children to select the picture that matched the spoken Korean word. After establishing the ceiling level for Korean receptive vocabulary skills, the examiner proceeded to assess their conceptual receptive vocabulary by introducing the English word for the incorrectly answered items to the children. To reduce the chance of children realizing their initial choices were incorrect and subsequently selecting different options, the examiner randomly questioned them about the items they had answered correctly. Likewise, the PPVT-4 assessment followed a similar approach to the REVT (receptive) task. In this case, the examiner initially provided words in English, followed by Korean words for the items that the children had answered incorrectly.

### 2.3 Procedures

A preliminary test was undertaken with three 5-year-old Korean–English bilingual children to assess the time required to complete a series of screening tests, parent–child shared book reading sessions, and early literacy tasks, as well as to determine the age-appropriateness of the books and early literacy tasks used in the study. Additionally, parental questionnaires were distributed to address any potential ambiguities or challenges for comprehension.

All tests in the experimental phase were conducted in quiet environments, either at the child’s home or in a private room at a library. The screening processes and early literacy tests took place in a one-on-one session between the child and the examiner. The parent–child shared book reading sessions occurred in a one-on-one setting, involving the child and the parent. The entire experiment unfolded in three sessions: (1) screening, which included Korean, English, and conceptual expressive and receptive vocabulary tests, as well as a nonverbal intelligence test; (2) Korean parent–child shared book reading and Korean early literacy tests; and (3) English parent–child shared book reading and English early literacy tests. During the vocabulary and early literacy skill tests, the examiner used either Korean or English, depending on the language being assessed. The nonverbal intelligence test was performed in the child’s preferred language. In cases where sessions were extended excessively or the child displayed signs of fatigue, the sessions were divided, except for the book reading session.

### 2.4 Data analysis

#### 2.4.1 The home literacy environment based on parental questionnaires and parent–child shared book reading

In assessing the aspects of the HLE through parental questionnaires, a 5-point Likert scale was employed, and the average rating for each section (PME, FL, and IL) was computed and used in the analysis. In terms of the quantity of available literacy resources, the actual numbers of Korean and English books possessed by the participants were considered for the analysis.

Regarding parent–child shared book reading, the initial 10 min of the book reading sessions were transcribed and analyzed. Excluded from the analysis were utterances that include habitual repetitions (e.g., phrases like “Isn’t it?” repeated after each sentence), self-talk, verbal mistakes, and any utterances unrelated to the context of the book reading (e.g., requests for the child to restate their previous statement or disciplinary utterances such as “Sit tightly” and “Focus”). The transcribed utterances were divided into text reading and interaction. Considering the flexibility of usage in morphemes in Korean, minor adjustments, such as changing, omitting, or adding case markers or alterations in negation within the book sentences, were deemed acceptable and regarded as text-reading utterances. The inclusion or omission of a single word within a sentence fell under the category of text-reading utterances as well. However, if a parent made significant changes to the overall sentence structure, it was considered an interactive utterance. Interactive utterances were categorized within each of the previously defined reading strategies, although not all interactive utterances were designated as the reading strategies. The Tr and the Kr were calculated by tallying the total number of words in each corresponding utterance. The extent of FLs and ILs was determined by counting the number of coded utterances. Average scores of parental sensitivity ratings on a 5-point Likert scale for each behavior were used for the analysis.

#### 2.4.2 Children’s early literacy and vocabulary skills

In the early literacy tasks, which encompassed SA, PA, LN, and OK, the percentage of correct responses was computed. For the vocabulary tests, which covered Korean, English, and conceptual expressive and receptive vocabulary, the raw scores were analyzed. In the stepwise multiple regression analyses, the combined scores of SA, PA, LN, and OK were utilized to represent early literacy skills. Similarly, the combined scores for expressive and receptive vocabulary were employed as indicators of vocabulary skills.

#### 2.4.3 Statistical analysis

All data analyses were conducted using *R* (version 4.3.0; R Core Team, 2023). Independent *t*-tests were employed to (1) assess the disparities between Korean and English HLEs measured through parental questionnaires, (2) compare the aspects of the HLE during parent–child book reading sessions between Korean and English book reading, and (3) explore differences in children’s early literacy skills in Korean and English. In the case of the number of books in the first analysis and the Korean interactive utterance ratio in the second analysis, Welch’s *t*-tests were applied due to unequal variances. Stepwise multiple regression analyses were performed to identify the HLE factors that predict children’s early literacy and vocabulary skills, controlling for age. Given the lack of prior research guiding the selection of specific HLE variables used in this study influencing these skills, forward stepwise multiple regressions were conducted. Variance inflation factors (VIF) for all the HLE variables were computed due to several correlations among HLE factors. The results showed that none of the VIF values exceeded the threshold of 5, indicating the absence of significant multicollinearity.

#### 2.4.4 Reliability

The assessment of PS involved two bilingual coders proficient in Korean and English. These coders independently rated parental sensitivity in each video of parent–child shared book reading sessions. The average ratings assigned by both coders for each behavior were computed, and the mean score across all assessed items was calculated for subsequent analysis. For consistency and accuracy, the second coder participated in two 90-min training sessions. In the first sessions, the coder was presented MULTI-PASS (Marfo, 1992) and each parental sensitivity behavior and examined examples of pre-coded outcomes from videos of book reading sessions recorded during the preliminary study for reference. In the second session, both coders viewed recorded videos of book reading sessions from the preliminary study together. The coders individually assessed the videos and compared their evaluations, collaborating to establish and refine the criteria used for coding parental sensitivity. The inter-coder agreement for ratings of parental sensitivity, calculated for a randomly selected 10% of the video recordings, stood at 85.4%, within the acceptable range of 85 to 90% (Miles et al., 2014).

For a randomly selected 10% of the video-recorded parent–child shared book reading sessions, an additional Korean–English bilingual second coder re-transcribed the book reading sessions and re-evaluated observed reading strategies. To maintain consistency, the second coder underwent a two-hour training session, which involved practicing transcription and coding reading strategies with the videos from the preliminary study. The inter-coder agreement between the first and second coders was found to be 95.8% for transcription and 86.0% for coding reading strategies.

## 3 Results

In this study, we examined the current HLEs of Korean**–**English bilingual children in Australia in both Korean and English. We compared the HLEs in each language and explored the differences in children’s early literacy skills between the two languages. In addition, we investigated the HLE factors in both languages that influence their Korean and English early literacy skills, as well as proficiency in Korean, English, and conceptual vocabulary.

### 3.1 Comparison of Korean and English home literacy environments and early literacy skills in Korean–English bilingual children

First, the results of independent *t*-tests and Welch’s *t*-test revealed that the number of Korean books was significantly greater than that of English books [*t*(65.17) = −3.03, *p* = 0.004], and the FL score in English was significantly higher than in Korean [*t*(70) = 2.48, *p* = 0.017]. However, no significant differences in PME [*t*(70) = −0.72, *p* = 0.480] and IL [*t*(70) = −1.18, *p* = 0.240] were found between Korean and English HLEs (Table 4).

**Table 4:** Results of *t*-tests comparing the Korean and English home literacy environments.

	Korean		English				
	*M*	SD	*M*	SD	df	*t*	*p*
Number of books	191.83	131.34	107.97	100.16	65.17	−3.03	0.004
Print and media exposure	3.27	0.68	3.15	0.76	70	−0.72	0.480
Formal literacy practice	2.80	0.94	3.36	0.99	70	2.48	0.017
Informal literacy practice	3.61	0.85	3.35	1.01	70	−1.18	0.240

In terms of HLE variables during book reading, a significant difference in Kr was observed between Korean and English book reading sessions [*t*(48.08) = −4.25, *p* < 0.001], indicating higher Kr during Korean book reading compared to English book reading. However, no significant differences were found in Tr [*t*(70) = −1.52, *p* = 0.133], FL [*t*(70) = 0.65, *p* = 0.515], IL [*t*(70) = 0.97, *p* = 0.334], and PS [*t*(70) = −1.54, *p* = 0.128] (Table 5).

**Table 5:** Results of *t*-tests comparing the home literacy environment factors between Korean and English book reading sessions.

	Korean book reading		English book reading				
	*M*	SD	*M*	SD	df	*t*	*p*
Text-read ratio	66.15	27.54	54.79	35.36	70	−1.52	0.133
Korean interactive utterance ratio	92.90	14.15	68.06	32.13	48.08	−4.25	0.000
Formal literacy practices	4.00	4.29	4.75	5.38	70	0.65	0.515
Informal literacy practices	50.81	18.49	55.64	23.40	70	0.97	0.334
Parental sensitivity	4.09	0.54	3.86	0.73	70	−1.54	0.128

A significant difference in LN was found between Korean and English early literacy skills, where children displayed greater knowledge of letter names in English than in Korean [*t*(70) = 9.65, *p* < 0.001]. However, no significant differences were observed between the two languages in SA [*t*(70) = −0.50, *p* = 0.621], PA [*t*(70) = 1.92, *p* = 0.059], and OK [*t*(70) = −1.05, *p* = 0.295] (Table 6).

**Table 6:** Results of *t*-tests comparing children’s Korean and English early literacy skills.

	Korean		English				
	*M*	SD	*M*	SD	df	*t*	*p*
Syllable awareness	88.82	14.99	81.94	16.97	70	−0.50	0.621
Phoneme awareness	55.19	22.39	65.19	21.89	70	1.92	0.059
Letter name knowledge	26.67	29.96	87.71	23.29	70	9.65	0.000
Orthographic knowledge	62.85	12.13	59.84	12.08	70	−1.05	0.295

### 3.2 Home literacy environment factors predicting early literacy and vocabulary skills in Korean–English bilingual children

To explore predictive factors in Korean and English HLEs, assessed through parental questionnaires and observed during book reading sessions, for children’s early literacy skills, combined scores of SA, PA, LN, and OK, as well as vocabulary skills, comprising expressive and receptive vocabulary scores, stepwise multiple regression analyses controlling for age were utilized.

First, models including general HLE factors measured by questionnaires that predict early literacy and vocabulary skills in children were created. The predictive model for Korean early literacy skills explained 59% of the variance [*F*(3, 32) = 17.69, *p* < 0.001], encompassing age [*β* = 0.73, *t*(32) = 6.60, *p* < 0.001], Korean FL [*β* = 0.32, *t*(32) = 2.89, *p* = 0.007], and Korean IL [*β* = −0.22, *t*(32) = −1.97, *p* = 0.058]. Korean FL had a significant positive impact on children’s early Korean literacy skills, while Korean IL had a non-significant negative effect.

For English early literacy skills, the model accounted for 41% of the variance [*F*(2, 33) = 13.26, *p* < 0.001]. Only age [*β* = 0.61, *t*(33) = 4.65, *p* < 0.001] and English IL [β = 0.23, *t*(33) = 1.77, *p* = 0.086] were included in the model, with English IL having a non-significant positive effect.

In terms of Korean vocabulary skills, a model including age [*β* = 0.22, *t*(31) = 1.81, *p* = 0.080], Korean PME [*β* = 0.63, *t*(31) = 5.03, *p* < 0.001], English PME [*β* = −0.33, *t*(31) = −2.56, *p* = 0.016], and the number of English books [*β* = 0.25, *t*(31) = 1.85, *p* = 0.074] accounted for 48% of the variance [*F*(4, 31) = 8.97, *p* < 0.001]. Age and the number of English books showed non-significant positive effects on Korean vocabulary skills. Korean PME significantly and positively impacted Korean vocabulary skills, while English PME had a significant negative impact.

A model predicting English vocabulary skills explained 59% of the variance [*F*(5, 30) = 11.27, *p* < 0.001]. It included age [*β* = 0.41, *t*(30) = 3.71, *p* = 0.001], Korean PME [*β* = −0.54, *t*(30) = −4.59, *p* < 0.001], English PME [*β* = 0.53, *t*(30) = 4.10, *p* < 0.001], and the number of Korean books [*β* = 0.41, *t*(30) = 3.00, *p* = 0.005] and English books [*β* = −0.24, *t*(30) = −1.78, *p* = 0.085] as predictive factors. Korean and English PME significantly influenced children’s English vocabulary, with Korean PME having a negative impact and English PME having a positive impact. The number of Korean books significantly and positively affected English vocabulary skills, whereas the number of English books had a non-significant negative impact.

Finally, the predictive model for conceptual vocabulary, which accounted for 66% of the variance [*F*(6, 29) = 12.37, *p* < 0.001], included five HLE factors in addition to age [*β* = 0.65, *t*(29) = 6.44, *p* < 0.001]. The number of Korean books [*β* = 0.53, *t*(29) = 3.95, *p* < 0.001] and English PME [*β* = 0.44, *t*(29) = 3.52, *p* = 0.001] had significant positive impacts on children’s conceptual vocabulary, while the number of English books [*β* = −0.26, *t*(29) = −2.15, *p* = 0.040] and Korean FL [*β* = −0.26, *t*(29) = −2.24, *p* = 0.033] had significant negative influences. Korean IL [*β* = 0.18, *t*(29) = 1.60, *p* = 0.121] had a positive effect, although it was not statistically significant.

Table 7 displays the results from the regression analyses investigating the HLE predictors assessed through parental questionnaires in relation to children’s early literacy and vocabulary skills.

**Table 7:** Results of regression analyses predicting children’s early literacy and vocabulary skills with HLE factors measured by parental questionnaires.

Dependent variables	Predictors	*B*	β	*t*	*p*	$R^{2}$	Adjusted $R^{2}$	*F*
KL	Age	7.19	0.73	6.60	0.000	0.62	0.59	17.69***
	K_FL	22.13	0.32	2.89	0.007			
	K_IL	−1.88	−0.22	−1.97	0.058			
EL	Age	5.01	0.61	4.65	0.000	0.45	0.41	13.26***
	E_IL	12.59	0.23	1.77	0.086			
KV	Age	1.17	0.22	1.81	0.080	0.54	0.48	8.97***
	K_PME	32.54	0.63	5.03	0.000			
	E_PME	−15.32	−0.33	−2.56	0.016			
	E_books	0.09	0.25	1.85	0.074			
EV	Age	2.27	0.41	3.71	0.001	0.65	0.59	11.27***
	K_PME	−29.69	−0.54	−4.59	0.000			
	E_PME	26.02	0.53	4.10	0.000			
	K_books	0.12	0.41	3.00	0.005			
	E_books	−0.09	−0.24	−1.78	0.085			
CV	Age	2.43	0.65	6.44	0.000	0.71	0.66	12.37***
	K_books	0.10	0.53	3.95	0.000			
	E_PME	14.22	0.44	3.52	0.001			
	E_books	−0.07	−0.26	−2.15	0.040			
	K_FL	−6.80	−0.26	−2.24	0.033			
	K_IL	5.34	0.18	1.60	0.121			

KL, Korean early literacy skills; EL, English early literacy skills; KV, Korean vocabulary skills; EV, English vocabulary skills; CV, conceptual vocabulary skills; K, Korean; E, English; FL, formal literacy practice; IL, informal literacy practice; PME, print and media exposure; books, number of books, ***p < 0.001.

Next, the study investigated how HLE factors observed during Korean and English book reading sessions predict children’s early literacy and vocabulary skills. The model predicting Korean early literacy skills accounted for 57% of the variance [*F*(3, 32) = 16.77, *p* < 0.001]. Age [*β* = 0.80, *t*(32) = 6.32, *p* < 0.001], PS during Korean book reading [*β* = −0.34, *t*(32) = −2.73, *p* = 0.010] and FL [*β* = 0.18, *t*(32) = 1.56, *p* = 0.129] during Korean book reading were included in the model. PS during Korean book reading exhibited a significant negative impact, while FL during KB had a non-significant positive influence.

The predictive model for English early literacy skills accounted for 42% of the variance [*F*(3, 32) = 9.57, *p* < 0.001], with age [*β* = 0.53, *t*(32) = 3.86, *p* = 0.001], Tr during English book reading [*β* = 0.23, *t*(32) = 1.79, *p* = 0.083], and FL during Korean book reading [*β* = 0.20, *t*(32) = 1.46, *p* = 0.155] as its components. Both HLE predictors showed non-significant positive impacts.

In the predictive model, which explained 33% of the variance in children’s Korean vocabulary skills [*F*(4, 31) = 5.26, *p* = 0.002], age [*β* = 0.16, *t*(31) = 1.03, *p* = 0.311], Kr during Korean book reading [*β* = 0.53, *t*(31) = 3.77, *p* = 0.001], PS during English book reading [*β* = 0.36, *t*(31) = 2.12, *p* = 0.042], and Tr during English book reading [*β* = 0.29, *t*(31) = 1.82, *p* = 0.079] were included. All variables had positive effects on Korean vocabulary skills, but only those of Kr during Korean book reading and PS during English book reading were statistically significant.

For English vocabulary, the predictive model accounted for 48% of the variance [*F*(4, 31) = 8.99, *p* < 0.001]. It included age [*β* = 0.47, *t*(31) = 3.44, *p* = 0.002], Kr during English book reading [*β* = −0.41, *t*(31) = −3.30, *p* = 0.002], PS during English book reading [*β* = −0.53, *t*(31) = −2.57, *p* = 0.015], and PS during Korean book reading [*β* = 0.30, *t*(31) = 1.42, *p* = 0.165]. Kr and PS during English book reading had a significant negative impact on English vocabulary skills, whereas PS during Korean book reading had a non-significant positive impact.

Lastly, no book reading HLE factors predicted conceptual vocabulary. A model with age [*β* = 0.69, *t*(34) = 5.49, *p* < 0.001] as the sole predictor explained 45% of the variance in conceptual vocabulary skills [*F*(1, 34) = 30.10, *p* < 0.001].

Table 8 provides an overview of the results from the regression analyses related to book reading HLE predictors and children’s early literacy and vocabulary skills.

**Table 8:** Results of regression analyses predicting children’s early literacy and vocabulary skills with HLE factors observed during book reading sessions.

Dependent variables	Predictors	*B*	β	*t*	*p*	$R^{2}$	Adjusted $R^{2}$	*F*
KL	Age	7.90	0.80	6.32	0.000	0.61	0.57	16.77***
	KB_PS	−41.46	−0.34	−2.73	0.010			
	KB_FL	2.80	0.18	1.56	0.129			
EL	Age	4.39	0.53	3.86	0.001	0.47	0.42	9.57***
	EB_Tr	0.36	0.23	1.79	0.083			
	KB_FL	2.55	0.20	1.46	0.155			
KV	Age	0.83	0.16	1.03	0.311	0.40	0.33	5.26**
	KB_Kr	1.33	0.53	3.77	0.001			
	EB_PS	17.41	0.36	2.12	0.042			
	EB_Tr	0.37	0.29	1.82	0.079			
EV	Age	2.64	0.47	3.44	0.002	0.54	0.48	8.99***
	EB_Kr	−0.48	−0.41	−3.30	0.002			
	EB_PS	−26.76	−0.53	−2.57	0.015			
	KB_PS	20.93	0.30	1.42	0.165			
CV	Age	2.55	0.69	5.49	0.000	0.47	0.45	30.10***

KL, Korean early literacy skills; EL, English early literacy skills; KV, Korean vocabulary skills; EV, English vocabulary skills; CV, conceptual vocabulary skills; KB, Korean book reading; EB, English book reading; FL, formal literacy practices; IL, informal literacy practices; Tr, text-read ratio; PS, parental sensitivity; Kr, Korean interactive utterance ratio, **p < 0.01, ***p < 0.001.

## 4 Discussion

This study aimed to examine the home literacy environment (HLE) and early literacy and vocabulary skills in Korean–English bilingual children living in Australia, where Korean serves as a heritage language in an English-speaking environment. In addition, the intricate relationships between the HLE and early literacy and vocabulary skills within this population were investigated. Korean and English HLEs were assessed through parental questionnaires, comprised of factors such as the number of books, print and media exposure (PME), formal literacy practice (FL), and informal literacy practice (IL). Additionally, parent–child book reading sessions, which involved reading one Korean book and one English book, were analyzed. The analysis included factors such as the text-read ratio (Tr), the Korean interactive utterance ratio (Kr), FL, IL, and parental sensitivity (PS). Children’s early literacy skills in both Korean and English, encompassing syllable awareness (SA), phonemes awareness (PA), letter name knowledge (LN), and orthographic knowledge (OK), were evaluated. Vocabulary skills, including Korean, English, and conceptual expressive and receptive vocabulary, were assessed.

### 4.1 Comparison of Korean and English home literacy environments and early literacy skills in Korean–English bilingual children

Participants had more Korean than English books, while they engaged in English FL more frequently than Korean FL. These results suggest that, with regard to Korean, parents provide an abundance of literacy resources, enabling more indirectly conducted literacy practices, whereas English literacy practices are focused on direct instruction, emphasizing letters themselves and related skills. These findings are consistent with previous research by Gonzalez-Barrero et al. (2021), which revealed that bilingual children tend to have a larger number of books in their dominant language, typically their native language, compared to their non-dominant language. Additionally, this aligns with a previous study conducted in Singapore, where English is the primary social language, and multiple heritage languages are spoken at home. It showed that Singaporean bilingual children generally receive more formal literacy instruction in English than in their home language (Sun et al., 2023).

Kr during parent–child shared book reading differed significantly depending on the language of the book. Parents tended to use more Korean interactive utterances when reading a Korean book compared to English, indicating an increase in the use of English for interaction during English book reading. These results suggest that bilingual parents adapt their language use based on the specific language context of the book they are reading (Gonzalez-Barrero et al., 2021; Quirk et al., 2022).

No differences in children’s early literacy skills were observed across languages, except for LN, where children demonstrated higher proficiency in English than in Korean. This disparity might be attributed to parents’ tendency to create a more formal literacy environment in English than in Korean, as earlier findings in this study indicated. It is widely acknowledged that FL strongly influences children’s code-related skills, including LN (Sénéchal et al., 2017). Therefore, the current results provide further evidence of variations in home-based literacy practices according to whether the language is a heritage or dominant social language.

### 4.2 Home literacy environment factors predicting early literacy and vocabulary skills in Korean-English bilingual children

First, predictive models were created for children’s early literacy and vocabulary skills using HLE factors measured by parental questionnaires. Korean early literacy skills were positively and significantly predicted by Korean FL, emphasizing the positive impact of frequent engagement in FL on children’s Korean literacy development. It suggests that a more active and instructional approach is essential to effectively promote literacy skills in a heritage language, especially when it is not the dominant language in society, rather than relying on indirect and passive methods. Although not reaching statistical significance, in terms of English early literacy skills, English IL was included in the predictive model. It implies that within the home setting, providing adequate exposure to the language is sufficient to foster the literacy development of English, the prevailing social language.

When it comes to Korean vocabulary skills, age did not play a significant role in Korean vocabulary skills, unlike other skills in children, which supports the idea that they may be experiencing subtractive bilingualism, where the development of Korean vocabulary decelerates even in their early childhood. The predictive model included Korean and English PMEs as significant factors, with Korean PME having a positive impact and English PME influencing negatively. Similarly, in the predictive model for English vocabulary skills, English PME had a significant positive impact while Korean PME had a significant negative impact. The time-on-task hypothesis, proposed by Rossell and Baker (1996), provides an explanation for the inversely-related outcomes observed in the cross-linguistic relationship between the home literacy environment and vocabulary skills. This hypothesis suggests that the amount of time dedicated to a specific task is directly linked to the level of achievement in that task. In the context of language learning, increased exposure and practice in a target language result in improved proficiency, which implies that, given limited time and resources, allocating more time to Korean literacy practices rather than to English would contribute to the development of Korean skills, and vice versa. Nonetheless, it is noteworthy that the number of Korean books at home had a significant positive impact on predicting English vocabulary skills. It is well-known that children’s language development can benefit from scaffolding, as suggested by Vygotsky (1978). In this context, books serve as supportive tools for language expansion, and the scaffolding effect can yield both direct and indirect advantages when parents engage in interactions while reading with their children (Hoff, 2010). It is possible that parents can offer more productive scaffolding when using books written in their native language, as they are most comfortable and confident in that language.

The predictive model for conceptual vocabulary revealed various contributing factors. Among them, the number of Korean books and English PME exerted positive influences, while the number of English books and engagement in Korean FL had negative effects. Substantial exposure to both Korean print and English print and media appeared to enhance the development of conceptual vocabulary in children. However, an excessive focus on letters alone may not be conducive to conceptual vocabulary skills. Instead, emphasizing the content of words is likely to be more advantageous. This is supported by the inclusion of Korean IL in the model as well, although it did not reach statistical significance. Regarding the negative impact of the number of English books, one plausible explanation is that parents may not be as effective in scaffolding their children’s vocabulary growth with English books as they are with Korean books. Thus, further research related to how parents utilize books not written in their native language is necessary.

Second, the study examined how HLE factors within the book reading context influence children’s early literacy and vocabulary skills. In the predictive model for Korean early literacy skills, interestingly, PS during Korean book reading had a significant negative impact. However, it would be inaccurate to conclude that higher levels of parental sensitivity during Korean book reading hinder the development of children’s Korean early literacy skills. When considering the components of PS, it is more closely associated with the content of the book and active interactions related to it rather than focusing solely on print. Therefore, to promote Korean early literacy skills, it appears that interactions that facilitate a focus on the print are necessary, which is supported by the inclusion of FL during Korean book reading in the model, despite its lack of statistical significance. In the model predicting English early literacy skills, the Tr during English book reading and FL during Korean book reading surfaced as predictors, yet not significant. These findings, which highlight the importance of implementing strategies that encourage children to pay attention to letters in print, are consistent with the previous studies that have documented a positive relationship between parents’ awareness of and engagement in formal literacy activities and children’s overall early literacy development (Anderson, 1995; Sénéchal et al., 1998).

Regarding Korean vocabulary skills, age did not emerge as a significant predictor, indicating the subtractive bilingualism environment that children are experiencing, and positive effects of Kr during Korean book reading and PS during English book reading were observed. The positive impact of Kr during Korean book reading on Korean vocabulary skills supports the effectiveness of parents’ scaffolding when using books in their native language and engaging in interactions in that language. The positive influence of PS during English book reading suggests the importance of actively involving children in interactive discussions and addressing their developmental needs while reading an English book to promote the development of Korean vocabulary skills. However, this study does not provide information on the level of children’s involvement during book reading sessions and the language children used for the interactions, indicating the need for further investigation.

As significant predictors for English vocabulary skills, Kr and PS during English book reading exhibited negative impacts. However, concluding that the use of Korean, the heritage language, and the higher levels of PS during English book reading have detrimental effects on children’s English vocabulary development is challenging since other factors may be at play. For instance, parents whose children have advanced levels of English vocabulary may adjust their language usage by employing more English in response to their children’s English competence, or children with strong English vocabulary skills may engage more actively in conversation while reading an English book without parental prompts. This study only analyzed parental interactive utterances without considering the interactive behaviors of the children, necessitating further research for more precise and comprehensive conclusions.

No significant HLE factors within a book reading context were found in the predictive model for conceptual vocabulary. This may be due to the limited sample size of the study or the possibility that the observed aspects of the HLE during book reading sessions do not fully capture the intricacies involved in conceptual vocabulary acquisition.

### 4.3 Limitations

First, the findings in this study need to be interpreted cautiously due to the small sample size of 36 parent–child dyads, which limits their generalizability. Additionally, the analysis focused solely on parents’ utterances during book reading sessions and did not consider how children react, respond, or initiate interactions, which restricts the full interpretation of the results. The analysis also only included the first 10 min of each book reading session, potentially missing important dynamics that may occur throughout the entire session. Therefore, it is suggested that future studies investigate more comprehensive and detailed aspects of interaction, including features of children’s interactive utterances. Furthermore, the study did not consider factors such as parents’ and children’s language proficiency in each language, the effects of siblings, parental beliefs and awareness of literacy education at home, and socio-economic status, including parental education levels, which are associated with literacy practices and available resources (Raz and Bryant, 1990; Dong et al., 2020). Future research should explore these additional factors to gain a more thorough understanding of the relationships between the HLE and children’s literacy skills in bilingual families.

## 5 Conclusion

In the current study, we aimed to uncover the relationships between the home literacy environment and literacy-related skills in Korean-English bilingual children residing in Australia, where the heritage language is Korean and English serves as a primary social language.

First, we examined the differences in the home literacy environment and children’s early literacy skills by language. The results indicated that parental literacy practices, in the case of Korean, were predominantly indirect and centered around books, while when it comes to English, these practices were more instructional and direct. As a result, children exhibited stronger familiarity with English letters compared to Korean. Furthermore, parents were observed to engage in more English language interactions during English book reading sessions, whereas they mainly used Korean when reading Korean books with their children.

In addition, the study investigated the predictors within the home literacy environment for children’s early literacy and vocabulary. To foster Korean early literacy skills, it is crucial to implement more active and explicit strategies that promote children’s direct interaction with written language, generally in a home setting and during book reading. While a somewhat indirect approach may be sufficient for developing English early literacy skills in a home environment, emphasizing the visual form of letters during English book reading can still be advantageous.

In line with the time-on-task hypothesis (Rossell and Baker, 1996), increased exposure to the Korean language had a positive effect on Korean vocabulary while negatively impacting English vocabulary skills. Similarly, increased exposure to the English language positively influenced English vocabulary but had a negative effect on Korean vocabulary skills. However, it is worth noting that access to Korean books at home remained a significant predictor of English vocabulary skills, suggesting that parents may provide more effective linguistic stimuli through books in their native language. A rich literacy environment, both in Korean and English, was found to support the development of conceptual vocabulary. Nevertheless, it is important to strike a balance, as an excessive focus on letters may not be as beneficial as emphasizing the content of words for the development of conceptual vocabulary. In the context of book reading, parents’ profuse and high-quality interactions appeared to contribute to the development of Korean vocabulary skills. However, this positive impact was not observed in relation to English or conceptual vocabulary skills. Further studies with a thorough investigation of parent–child interactions during parent–child shared book reading are necessary.

This study provides insights into conducting research on the bilingual home literacy environment by highlighting the importance of taking both languages into account when exploring the relationships between the home literacy environment and children’s foundational skills for subsequent literacy development. In addition to utilizing questionnaires to assess the overall home literacy environment, this study also analyzed parental literacy practices during actual book reading activities. This approach has practical implications for offering constructive and detailed guidance to parents and educators on how to effectively nurture early literacy and vocabulary skills in bilingual children through a home literacy environment in a comprehensive manner.

Furthermore, the results of this study provide empirical evidence underscoring the critical significance of establishing an enriched heritage language home environment to foster literacy and language proficiency in both languages, of which implications extend beyond the individual level at home to educational settings (Giambo and Szecsi, 2015; Andreou et al., 2020). These findings are expected to raise awareness among bilingual families and educators regarding the cultivation of literacy skills within a heritage language and the necessity of creating an environment conducive to this goal.

## Data availability statement

The raw data supporting the conclusions of this article will be made available by the authors, without undue reservation.

## Ethics statement

Ethical approval was not required for the study involving human samples in accordance with the local legislation and institutional requirements. Written informed consent for participation in this study was provided by the participants’ legal guardians/next of kin.

## Author contributions

W-JK: Conceptualization, Data curation, Formal analysis, Investigation, Methodology, Resources, Validation, Visualization, Writing – original draft, Writing – review & editing. DY: Conceptualization, Funding acquisition, Project administration, Supervision, Writing – review & editing.
